# Hepatic Abscess

**Published:** 1908-05-09

**Authors:** William Turner

**Affiliations:** Surgeon to Out-patients, Westminster Hospital; Surgeon to the Seamen's Hospital


### HEPATIC ABSCESS.

By WILLIAM TURNER, M.S., M.B., F.R.C.S. Surgeon to Out-patients, Westminster Hospital; Surgeon to the Seamen's Hospital.

Abstract of a Lecture delivered at the London School of Clinical Medicine, Seamen's Hospital, Greenwich.

Abscesses of the liver are caused by various micro-organisms and sporozoa, among the latter particularly the amœba coli. The organisms may reach the liver directly by trauma, or indirectly by the hepatic artery, by the portal vein, and by the bile ducts. Excluding altogether the traumatic cases, and confining our attention solely to the others, it is first to be noted that the condition may be single or multiple. Such examples of multiple abscess as are due to gall-stones, and to portal or systemic pyæmias are in most cases beyond help surgically. But single, even double and triple, abscesses are different. The single abscess *par excellence* is the "tropical abscess"; but do not forget that tropical abscesses may be multiple also. Such abscesses contain no bacteria; they are caused by the amœba coli, and even that is often not found in the pus until the third day after the abscess has been opened.

This tropical abscess has peculiar relations to dysentery, and many cases in which there was no history of this connection have been proved to be examples of it by post-mortem examination. It nearly always occurs in men, though women do occasionally suffer, and they have mostly resided in the tropics. They are commonly those who have led an active life. The origin of the condition is probably by embolism through the portal vein of infective material from the enteric lesions of dysentery; that is, of amœbic dysentery. In the South African war dysentery was very common, liver abscess very rare, because the dysentery was not amœbic, but bacillary: the post-mortem appearances, indeed, were frequently suggestive of enteric fever rather than of dysentery. But in the actual tropics dysentery is usually caused by the amœba.

Why people get liver abscess is a question which is difficult to answer. During and just after dysentery, hepatitis is common, and it is also excited, apart from dysentery, by cold, exposure, and alcoholic intemperance. It seems that when anyone who has ever had dysentery gets hepatitis he is especially liable to develop an abscess. The prognosis of tropical abscess depends largely on whether the abscess is single or multiple: it is generally said that the proportion is three cases of the former to every one of the latter.

There are three main periods during which a patient may develop hepatic abscess: (1) Directly following dysentery. In such cases when the dysenteric symptoms clear up the temperature does not come down, and the signs and symptoms of abscess supervene almost straightaway. (2) Some considerable time after an attack. Attacks of hepatitis may clear up, or sometimes, when the subject has previously had dysentery, may go on to form an abscess. (3) The very late cases. Many years after residence in the tropics has ceased, a liver abscess may form. I have quite lately seen cases in which the intervals between the attack of dysentery and the formation of abscess have been twenty-eight and seventeen years. In the former case the patient had no intestinal trouble whatever during the interval.

In text-books the symptoms of liver abscess may appear characteristic; there is no time to mention all of them, but the great point is that they are nearly always anomalous. The diagnosis is generally extremely difficult, and not a simple matter at all. I will read out two or three histories from notes of actual cases, and it will be seen what great differences there are. A patient had dysentery in June; in November of the same year "fever" for ten days. In January following was "quite well," but in February had a nocturnal temperature of 100 to 101 deg. F. She then complained of pain in the loin and in the shoulder, and is said to have presented "evidences of hepatitis." In April the patient was not improving, and had pain in the hepatic region and down the right arm. In May, without any warning, she suddenly coughed up between two and four ounces of typical liver pus, and again at the beginning and at the end of July. At an operation towards the end of August an empyema was found at the right base, full of liver pus.

Another case was admitted on a Wednesday into hospital, having been taken suddenly ill on the previous Saturday. There was no rigor, no vomiting, and no history of dysentery. This patient had left Bombay four years previously. He had a temperature of 103 deg. F., but no physical signs to account for it. There were no parasites in the blood. The temperature was of a high remittent character for several days. A diagnosis of peritonitis, probably arising from appendicitis, was made, but the case eventually turned out to be one of liver abscess.

Yet another case was that of a surgeon on one of the P. and O. boats, who while at sea suffered from severe diarrhœa, not dysenteric in character, for four days from August 15, 1898. He was then well until August 24, when he had vomiting and diarrhœa for one day. On October 11 he felt ill, and his temperature was 102 deg. F. He took quinine, but was admitted to hospital on October 14 with severe pyrexia, due to multiple abscesses, five of which were opened at operation. A great number of others were found post mortem, and the case was, in fact, one of portal pyæmia, due to the amœba coli.

Of the various symptoms, wasting is one of the most constant, and is often very striking. Sweats, too, are frequent, and are mistaken in many cases for those of malaria. What are the chief things to look out for in making the diagnosis? (1) The fever in nearly every case is hectic; it may be regular or irregular. (2) Wasting is apparent in almost every case. (3) There is no enlargement of the spleen. (4) Examination of the blood shows a high leucocytosis, due to increase of the polymorphonuclear cells. (5) There is a history of residence in the tropics nearly always, and of dysentery in 75 per cent. of cases. (6) The physical signs disclosed by examination of the liver, which is often very difficult, are most important.

The seat of election of a tropical abscess is in the upper part of the right lobe, close up under the diaphragm. This site is surrounded in every direction by at least four inches of tissues, and it is an abscess in this situation which causes the greatest difficulty in diagnosis. When an abscess here has attained a certain size it may be noticed that the upper edge of the liver dullness no longer descends gradually as it is traced from the sternum to the axillary line, but either is level or actually rises: that is, the liver dulness in the axilla is higher than normal. At the back the dulness either runs upwards right to the spine, or downwards from the uppermost limit in the axilla, with resonance between the top of the curve and the spine. A level line of dulness behind is more suggestive of empyema than of abscess, and this sign was actually found in the first of the cases which I mentioned just now.

In cases where the abscess is otherwise placed, there may be little or no alteration of the upper limit of hepatic dulness, but a bulging of the lower limit downwards, even as far as the iliac fossa. Such abscesses are more anterior in the liver, and consequently easier to diagnose. Occasionally an abscess of the left lobe of the liver may simulate very completely an enlarged spleen. In one case the dulness due to an abscess in this lobe filled the whole left hypochondrium, and seemed quite distinct from the apparently normal liver dulness.

It is uncommon to find malarial organisms in the blood, though they do occur sometimes.

Pain in the subjects of hepatic abscess is very common in the shoulder and down the arm, and is sometimes almost excruciating. When complaint of pain is greatest, the abscess has generally been at the seat of election, and has perforated the diaphragm into the lung. The explanation of the pain is uncertain, but it has been supposed to be a reflex effect of irritation of the phrenic nerves. This is somewhat questionable, though it is not easy to find grounds on which to dispute it.

An abscess at the seat of election is four inches deep from every surface, and its treatment is entirely surgical. Any suspected case of tropical abscess ought to be explored under an anæsthetic. I say under an anæsthetic for two reasons. Firstly, because if pus is found, one can proceed to operation at once. Secondly, because it may be necessary to plunge a needle or trocar very deeply, and the slightest movement of the patient may lacerate his liver a great deal, and set up extensive hæmorrhage.

Abscesses which point abdominally may be opened in one stage or in two. That is, one may do a preliminary operation to shut off the peritoneal cavity by sutures, and complete the draining of the abscess after adhesions have formed; or the operation may be completed at one time. When suturing off the peritoneal cavity great care is required, as it is very difficult to get stitches to hold in the liver.

Abscesses at the seat of election must often be opened from behind. When an abscess is confined to the liver, opening the pleura is a very safe and simple undertaking. But if the lung is already involved in the abscess, and from it the pleura be infected during drainage of an abscess, a pyopneumothorax is extremely likely to follow, and this is the great danger of the operation. If, therefore, the pleura is opened, it ought to be shut off from the field of operation before proceeding; for if it is not thickened and shut off by adhesions, the layers can be stitched together all the way round the incision; one can then wait forty-eight hours for adhesions to form, and after that proceed with the operation.

There is time to mention but two more points to-day. Here is Sir Patrick Manson's trocar or cannula, which is used for draining liver abscess when other treatment is impossible. It is a device for introducing a drainage tube into the track of a metal cannula, and, though somewhat rough and ready, it is an instrument which has been extremely successful. Cantlie's instrument is of a different shape, but is the same in principle, and is used in similar cases.

The success of operations and their after treatment depends on rigidity and strictness of antiseptic observance. Every dressing must be carried out with the utmost antiseptic precaution, for if once the wound is infected with pyogenic organisms, the result is disastrous.

